# The Application of Polysaccharides and Their Derivatives in Pigment, Barrier, and Functional Paper Coatings

**DOI:** 10.3390/polym12081837

**Published:** 2020-08-16

**Authors:** Qianlong Li, Shanyong Wang, Xuchen Jin, Caoxing Huang, Zhouyang Xiang

**Affiliations:** 1 State Key Laboratory of Pulp and Paper Engineering, South China University of Technology, Guangzhou 510640, China; 201730440191@mail.scut.edu.cn (Q.L.); feshanyong@mail.scut.edu.cn (S.W.); fexcjin@mail.scut.edu.cn (X.J.); 2 Jiangsu Co-Innovation Center of Efficient Processing and Utilization of Forest Resources, Nanjing Forestry University, Nanjing 210037, China; hcx@njfu.edu.cn

**Keywords:** polysaccharide, derivatives, modification, paper, coating, function

## Abstract

As one of the most abundant natural polymers in nature, polysaccharides have the potential to replace petroleum-based polymers that are difficult to degrade in paper coatings. Polysaccharide molecules have a large number of hydroxyl groups that can bind strongly with paper fibers through hydrogen bonds. Chemical modification can also effectively improve the mechanical, barrier, and hydrophobic properties of polysaccharide-based coating layers and thus can further improve the related properties of coated paper. Polysaccharides can also give paper additional functional properties by dispersing and adhering functional fillers, e.g., conductive particles, catalytic particles or antimicrobial chemicals, onto paper surface. Based on these, this paper reviews the application of natural polysaccharides, such as cellulose, hemicellulose, starch, chitosan, and sodium alginate, and their derivatives in paper coatings. This paper analyzes the improvements and influences of chemical structures and properties of polysaccharides on the mechanical, barrier, and hydrophobic properties of coated paper. This paper also summarizes the researches where polysaccharides are used as the adhesives to adhere inorganic or functional fillers onto paper surface to endow paper with great surface properties or special functions such as conductivity, catalytic, antibiotic, and fluorescence.

## 1. Introduction

Polysaccharides, as one of the most abundant natural polymers, widely exist in animals, plants, and microorganisms. Different from oligosaccharides, polysaccharides are usually composed of 10 or more monosaccharides connected by glycosidic bonds. The most common polysaccharides include cellulose, hemicellulose, pectin, starch, chitosan, sodium alginate, etc. Based on their different categories and sources, polysaccharides have different monosaccharide compositions and glycosidic linkages, which also give them unique biological activities and functional characteristics. For instance, the amino groups are responsible for the antibacterial properties of chitosan [1], and the sulfate groups are the major reason for the antioxidant property of sodium alginate [2,3].

Coating is a key step in paper production, which is used to improve the performance or function of paper. For packaging paper, paper coating is mainly a barrier coating, in which polymers are often applied to the paper surface to improve its mechanical property, hydrophobicity, or permeability (such as oxygen or water vapor permeability [4]). For printing paper, paper coating is mainly pigment coating, in which inorganic fillers such as calcium carbonate are filled into the pores between fibers on paper surface to improve its weight, whiteness, glossiness, and flatness for better printing and optical properties [5,6,7]. However, inorganic fillers cannot adhere to the paper surface stably, so using adhesives is necessary, which enable the inorganic filler to be fixed on the paper surface. In addition, paper coatings also include functional coatings, in which inorganic fillers are replaced by some functional fillers, and adhesives are also needed to make the fillers firmly attached to the paper surface. By using special fillers, i.e., metal nanoparticles and carbon nanotubes (CNTs), paper with antibacterial, conductive, catalytic, fluorescent, and other special functions can be prepared. The polymers or adhesives used in barrier, pigment, and functional coatings are mainly petroleum-based synthetic polymers, including polyvinyl alcohol (PVA) [8], styrene butadiene latex [4], polyethylene terephthalate [4], acrylic acid [9], vinyl acetate [10,11], and so on. Petroleum-based polymers have good mechanical, hydrophobic, and barrier properties, and are well applied in paper coatings. However, these petroleum-based polymers are difficult to be naturally degraded [12] causing serious environmental pollution. With energy shortage and environmental problem increasing prominently, searching for a renewable and biocompatible polymer to replace the petroleum-based polymers used in paper coating is a pressing task [12].

Among the various types of natural polymers, polysaccharide has the potential to replace petroleum-based polymers in the paper coating industry. Polysaccharides have strong water binding ability because of their abundant hydroxyl groups, and can easily be dispersed or dissolved in water. They also have good film-forming properties [13]. The raw material of paper is mainly plant fibers, which contain mostly polysaccharides, i.e., cellulose. The hydroxyls of polysaccharide can link with the hydroxyls of paper fibers through hydrogen bonding, resulting in high affinity of polysaccharides to paper surface [14]; the long chain structure enables their molecules to easily tangle with each other, forming mechanically reinforced network [15]. In addition, some polysaccharides have amphiphilic chemical structures which contribute to good dispersing ability to inorganic fillers, and thus the fillers can be dispersed and adhered to the paper surface more uniformly, improving the coating performance. However, polysaccharides do not behave well as a good moisture barrier because of their natural hydrophilicity [16]. The poor mechanical property of pure polysaccharide coating layers also limits their application in pigment and functional coatings. The properties of polysaccharides can be improved via physical or chemical methods to meet the requirements of applications in paper coatings. The abundant active hydroxyl groups of polysaccharides make prompt chemical modifications on polysaccharides possible.

**Figure 1:**
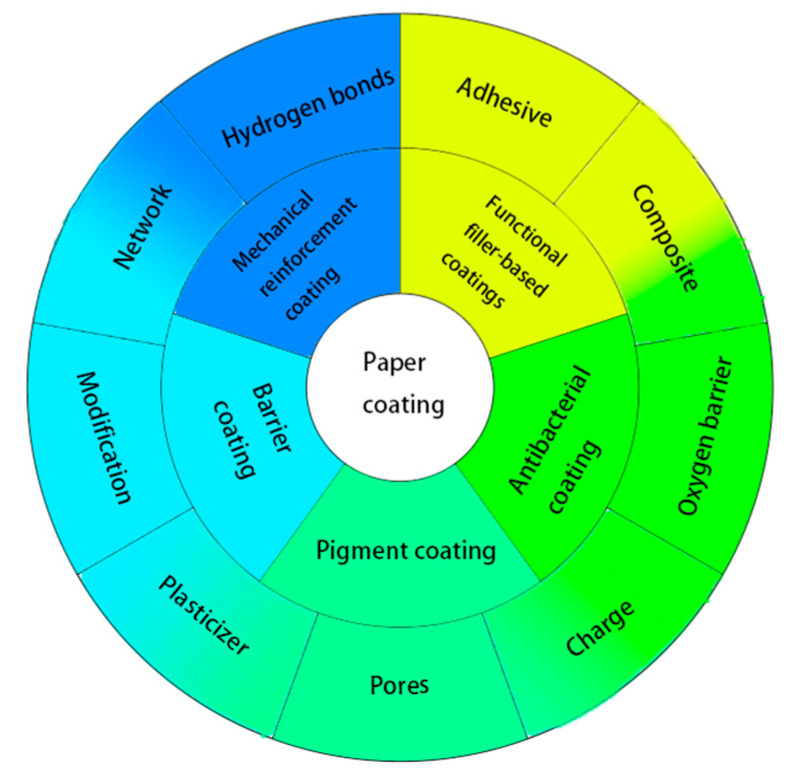
Factors affecting the properties of different types of paper coatings.

This paper reviews the research progress of paper coatings based on natural polysaccharides and analyzes the influencing factors of polysaccharides in coatings, such as hydrogen bonds, charges, network structures, plasticizers, modifications, and so on (Figure 1). These factors may endow coated paper with mechanical reinforcement, barrier property enhancement, antibacterial, and other functional effects. The advantages of the unique structures and functional groups of polysaccharides and their derivatives in coatings were discussed.

## 2. Mechanical Reinforcement Coating

**Figure 2:**
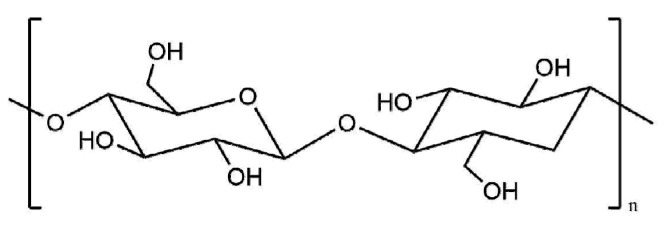
Chemical structure of cellulose.

Cellulose is the most abundant natural polysaccharide in nature. It is a linear polymer (molecular weight ranging from tens of thousands to hundreds of thousands [17]) formed by d-glucopyranose via β-1,4-glucosidic bonds (Figure 2). Although cellulose has long molecular chain and high hydroxyl content, the solubility of cellulose in general solvent is limited [18]. The solubility of cellulose can be improved by chemical modification resulting in better applications in paper coating. Cellulose based mechanical reinforcement paper coatings mainly include cellulose etherification or esterification products, such as carboxymethyl cellulose (CMC) and hydroxyethyl cellulose (HEC).

The CMC molecule has negative charges resulting in stable electrostatic repulsion between CMC molecules. The whiteness and printing performance of paper will be improved with the increase of CMC content in the coating [19]. It is worth mentioning that the filler retention by the addition of CMC has been improved because of the electrostatic binding effect between CMC and the filler with charges, i.e., precipitated calcium carbonate [20].

Nanocellulose is obtained from cellulose fibers by nano-fibrillation. There are three types of nanocellulose. Cellulose nanofibrils (CNF) with a diameter of 10–100 nm and a high aspect ratio can be obtained by separating the microfibrils of cellulose fiber through extensive mechanical treatment. By hydrolyzing the amorphous area of cellulose fiber bundle, the short rod-shaped crystalline nanoparticles can be obtained and are known as cellulose nanocrystals (CNC). Because of its large specific surface area, good chemical reactivity, and good rheological properties, nanocellulose has unique performance in paper coatings [21]. The large aspect ratio of CNFs makes them more tightly entangled, which can better enhance the mechanical properties of the coating layer. Moreover, the density of the CNF fiber network can be further improved through esterification crosslinking (acrylates), thus greatly increasing the mechanical properties of the coating layer [22,23]. In paper coating, the hydroxyl group of CNF can be closely bonded with the paper surface fiber through hydrogen bond [24], and CNF can also intertwine with the paper fibers to further improve the mechanical properties of the coated paper [15]. CNCs are short rod-shaped particles and also have a large number of hydroxyl groups which can form a dense coating layer on paper surface [25,26]. CNC is usually used as an additive in paper coating, which can better enhance the rigidity and toughness of the coated paper [27]. However, unlike that the high aspect ratio CNF can intertwine with paper fiber, there is little help to enhance the mechanical properties by deposition of particle like CNC on the paper surface, because a stacked structure forms. CNC may even decrease the mechanical properties of coated paper after water treatment [28]. Bacterial cellulose (BC) is a kind of natural nanocellulose secreted by specific bacteria and has unique physical, mechanical properties and high purity [29]. The most studies of BC focus on biomedicine and reinforcement in nanocomposites. There are very few studies applying BC in paper coatings [30], but with a few studies using BC to reinforce or prepare functional paper composites, e.g., catalytic paper [31], paper electrode [32], and fluorescent paper [33]. However, BC has been proved that it can be used as adhesive [34] which has the potential to be a paper coating adhesive.

**Figure 3:**
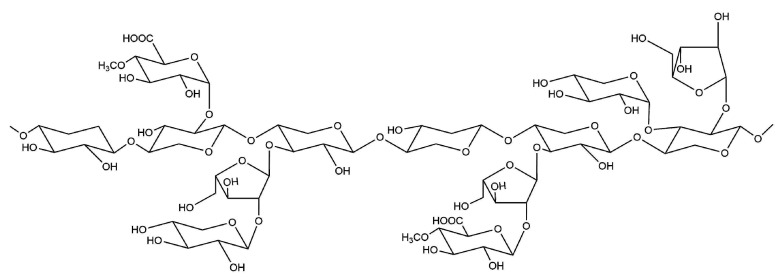
Chemical structure of arabinoxylan.

Hemicellulose is a natural abundant polysaccharide with branched structures (Figure 3). It is formed by condensation of some monosaccharide and uronic acid elements such as d-xylose, d-glucose, l-arabinose, d-galactose, d-mannose, d-glucuronic acid, and d-galacturonic acid [35,36]. Both the main and branched chains have many hydrophilic groups (such as hydroxyl, carboxyl, etc.,). Compared with cellulose, hemicellulose has smaller molecular weight, stronger water dispersibility and hydrocolloidal property, so it has the potential to prepare paper mechanical reinforcement coatings. The abundant hydroxyls of hemicellulose confer a strong affinity between cellulose and hemicellulose. Besides, the straight molecular structure of hemicellulose are easier to form hydrogen bonds with cellulose, compared with the helices chain of starch [37]. However, when unmodified hemicellulose is used in paper coating, the improvement in paper mechanical property is limited [38,39], so it needs to be properly modified physically or chemically. The xylan esterified by (2-dodecen-1-yl) succinic anhydride can significantly improve the mechanical properties of coated paper because of the plasticizing effect of the long aliphatic chains of DSA and tight interaction between cellulose and xylan via hydrogen bonding. The burst resistance was 70% and the tear strength was 60–80% higher than the base paper [40].

**Figure 4:**
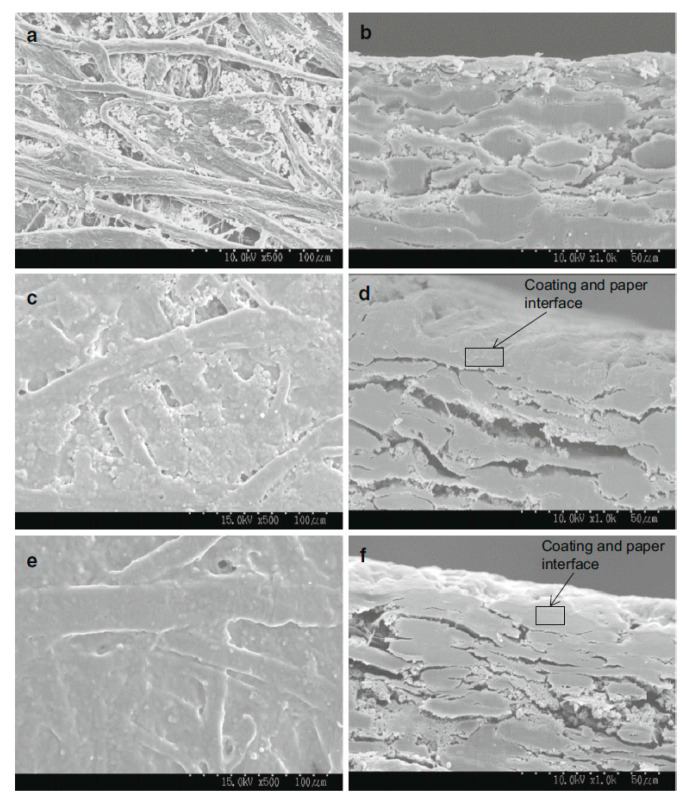
SEM images of paper surface (**a**,**c**,**e**) with magnitude ×500 and cross-sections (**b**,**d**,**f**) with magnitude ×1000 for uncoated paper and paper coated with AX and GAX with coating weight of 12 g/m^2^. (**a**,**b**) uncoated paper; (**c**,**d**) unmodified AX coated paper; (**e**,**f**) GAX coated paper [6] (Reprinted by permission from: Springer, Cellulose, Glutaraldehyde crosslinking of arabinoxylan produced from corn ethanol residuals, Xiang, Z.; Anthony, R.; Lan, W.; Runge, T., 2016).

In addition to hydroxyl functionalization, cross-linking reaction can enhance the interaction between polysaccharide molecules, making the network between polysaccharide molecules stronger and further improving the mechanical properties of coating layers [41]. In addition, if polysaccharides can form covalent bond with the paper fiber, it would further improve the mechanical properties of the paper. Xiang [6] and Hu [42] extracted arabinoxylan from distiller’s grains and sugarcane bagasse respectively, crosslinked it with glutaraldehyde to obtain glutaraldehyde crosslinked arabinoxylan (GAX), and applied it to paper coating. The SEM picture of coated paper is shown in Figure 4. The coatings can almost cover the entire paper fibers (Figure 4a,c,e). From the paper cross-sectional pictures (Figure 4b,d,f), it showed that the GAX coating had higher affinity to paper surface than unmodified AX coating [6]. This suggested that the aldehyde groups of GAX formed covalent bonds with the paper surface fibers in the form of acetal or hemiacetal, which improves the dry strength by 25% and the wet strength by 90% of the paper. The paper performance after using the GAX coatings was comparable to that coated with PVA.

In addition, there have been few studies on the effects of polysaccharide viscosity on paper coating. Polysaccharides with high viscosity have strong intermolecular interaction, large molecular volume, and slow movement, so they are more difficult to penetrate into the paper fiber matrix [43] which affects the uniformity and effect of coating, so it is necessary to increase the coating time and the amount of coating for high viscous polysaccharides. In addition, viscous polysaccharide coating can hardly be adapted to high-speed machine coating. Xiang reduced the intermolecular interaction of xylan by succinylation, thus reducing the viscosity of xylan suspensions [44]. However, the mechanical properties of paper coated by the succinylated xylan coating are comparable to those coated by PVA coating, having a good mechanical enhancement effect [45]. The mechanical properties of different polysaccharide-coated paper are summarized in Table 1.

## 3. Barrier Coating

Polysaccharides can be applied to prepare dense coating layers on the surface of paper by intertwining or chemical binding between polysaccharide molecular chains and paper fibers. The dense coating layer and functional groups on polysaccharides can impede the penetration of oxygen, water, or oil. The barrier performance is mainly divided into gas barrier, water barrier, and oil barrier. Gas barrier includes water vapor and oxygen barrier performance. Generally, gas barrier performance is improved because of the decrease of porosity. Water barrier is the hydrophobic effect of the paper-coating surface to liquid water, which prevents the water from penetrating into the interior of the paper and causing the structure of the paper to be destroyed. Through modification and other methods, the hydrophobicity of the coating surface can be effectively improved. For example, the entangled structure among CNF fibers and their high crystallinity enable the formation of a dense layer on the paper surface, providing good oxygen barrier properties [14]. The hydroxyl groups of polysaccharide structure provide oil resistance but weaken water resistance [48].

### 3.1. Gas Barrier

**Figure 5:**
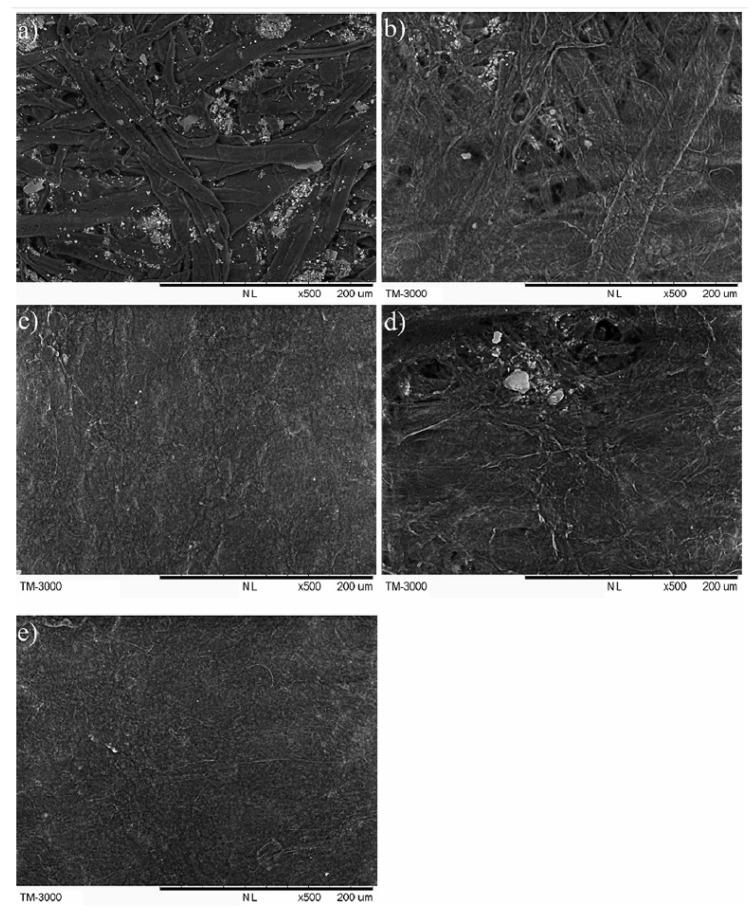
SEM images of CNF-coated paper:(**a**) uncoated base paper; paper coated with 2% (**b**) rCNF and (**c**) gCNF along with CMC, respectively; paper coated with 3% (**d**) rCNF and (**e**) gCNF along with CMC, respectively [49] (Reprinted by permission from: Springer, Cellulose, Cellulose nanofiber/carboxymethyl cellulose blends as an efficient coating to improve the structure and barrier properties of paperboard, Mousavi, S.M.M.; Afra, E.; Tajvidi, M.; Bousfield, D.W.; Dehghanifirouzabadi, M., 2017).

The coating with better gas barrier properties can be obtained by compositing cellulose with other matrix materials. For example, Mousavi [49] applied CMC/CNF composite coating onto paper surface and found that the electrostatic repulsion due to CMC surface charges reduced the CNF flocculation, improving its dispersibility; comparing Figure 5a–e, it shows that a uniform and compact coating layer on paper surface was thus formed and able to block the pores between paper fibers, giving the paper higher water vapor resistance and effectively reduced surface roughness.

Through corona discharge, Mirmehdi improved the adhesion of coating on paper surface [50]. Through spray coating method, the pores of CNF fiber network were filled with nano clay, producing uniform and dense coating layers, which decrease the water vapor transmission rate by 86% and decrease the oxygen transmission rate by 90%. CNC with short-rod shapes can form a dense network on the paper surface and a tortuous path. Since the air and water are difficult to pass through the coating layer, the water and air permeability of the coated paper can be reduced [28]. With extensive homogenization of CNC particles in coatings, the barrier properties of coatings can be further improved; for example, the oxygen barrier performance can be improved by 98% [51].

Physical modification, such as addition of plasticizer (glycerol, sorbitol, xylitol, etc.,), can effectively improve oxygen barrier performance and water vapor permeability of hemicelluloses [52,53]. Galactoglucomannans (GGM) can be plasticized with sorbitol, and the gas barrier performance of coated paper is substantially improved. However, a large amount of plasticizers are usually needed, and the migration of plasticizers limits its application in food packaging paper [54]. Chemical modification can also effectively improve the barrier performance of the coating. A suitable degree of substitution (DS) of hydrophobic functional groups can provide good hydrophobicity [6]. Polysaccharide derivatives with long-chain alkyl can form a dense accumulated structure in the interior of coatings, and prevent the passage of water molecules and gas molecules. Kisonen [55] applied O-acetyl GGM esterified with benzoic anhydrides to coat paper for food packaging. The hydrophobic property of the phenyl group effectively decreased the water vapor permeability of the packaging paper by 81% and the anti-grease property was also slightly improved because of the natural hydrophilic property of mannans, thus extending the shelf life of packaged food. Ramos [40] applied carboxymethyl xylan and dodecenyl succinic anhydride (DSA) modified xylan to the coating of cardboard and packaging paper. It was found that the mechanical properties of coated paper have been significantly improved because of the plasticizing effect of anhydride. The long aliphatic carbon chains of DSA replacing the hydroxyl groups of polysaccharides decrease the polarity and hydrophilicity making the water vapor permeability reduced by 30-fold when compared to the base paper. Its gas barrier performance is also comparable to the traditional PVA-coated packing paper. Arabinoxylan coating has good gas barrier property, but its hydrophilicity resulted in low water vapor barrier property and low oxygen barrier property under high humidity condition. Grondahl [56] enhanced hydrophobicity of hemicellulose and reduced its hydrophilicity in wet air by trifluoroacetic anhydride modification. The contact angle of surface increased from 30° to 70°.

**Figure 6:**
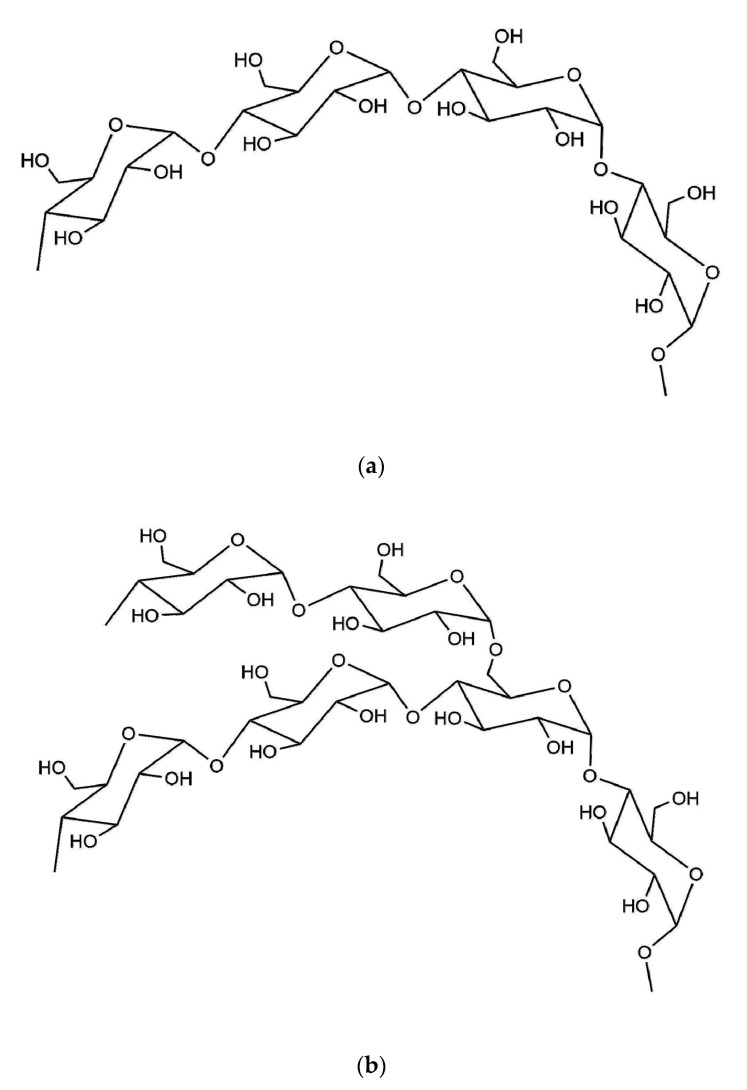
Chemical structure of (**a**) amylose and (**b**) amylopectin.

Starch is also a promising polysaccharide applied as barrier coatings. The main chain structure of starch consists of d-glucopyranose and is connected by α-1,4-glucoside bond [18], which determines its natural spiral structure. According to whether starch has branched chains, starch can be divided into amylose (Figure 6a) and amylopectin (Figure 6b). Amylose does not have, while amylopectin has abundant branched chains. The majority of natural starches are amylopectin, which has the film-forming ability, but its film mechanical properties still need to be improved [57]. Being used as paper coatings, pure starch still has some other drawbacks. For instance, starch is sensitive to water vapor and usually forms a brittle coating layer [58]; pure starch will also form faults in coating layers because of residual air, resulting in large surface pores. Consequently, it is usually modified by gelatinization and etherification. The hydroxypropyl starch coating can effectively improve the barrier property of coated paper, but because of the sensitivity of starch to water vapor, the moisture uptake of starch coating below 50% relative humidity was higher than CMC. Latex and other emulsions are usually added to squeeze out the air and fill in the faults to improve the barrier performance of the starch coating. The latex addition to the hydroxypropyl starch can reduce the porosity of coating which can be used in food packaging paper to block the penetration of mineral oil and improve the safety performance of packaging paper. With the content of latex increase, the water barrier property is improved while the oxygen and mineral oil transmission rates are high due to the low polarity of latex [59]. Garcia proposed to prepare barrier coatings with different proportions of ester (sunflower oil), starch, and plasticizer (sorbitol, glycerin) [60]. Sunflower oil was added to starch suspensions after adding sorbitol and glycerin as plasticizers. Plasticizers and esters play an important part in improving the anti-moisture permeability of the coating, enhancing the mechanical properties. Crystalline structures are responsible for extremely tightly packed structure and tend to be impermeable. However, plasticizers such as glycerol and sorbitol will hinder the polymer chains from aggregating which decreases the crystallinity of coating layers and weakens the water resistance.

Starch nanocrystals (SNCs) and starch nanoparticles (SNPs) are both starch products with nano-size. SNC is produced by hydrolyzing the amorphous part and leaving the crystalline part of starch, while SNP is the starch nanoparticles with both crystalline and amorphous parts [61]. SNPs has been commercialized as an adhesive (Eco-sphere TM) and can be used in paper coating to substitute PVA. Cassava starch-based films with 2.5% wt SNPs addition can have water vapor permeability reduced by 40% [62]. The platelet-like low permeable structure of SNCs can also reduce oxygen diffusion and permeability of starch-based films [61].

**Figure 7:**
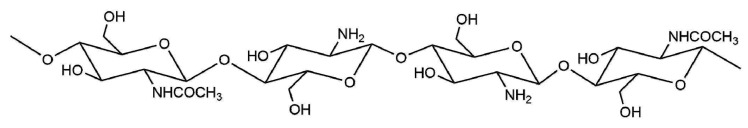
Chemical structure of chitosan.

Chitin is composed of *N*-acetylglucosamine connected by β-1,4-glucoside bond. Chitosan is a product of deacetylation of chitin, and its water solubility is affected by the degree of deacetylation (Figure 7). The higher the degree of deacetylation, the better is the water solubility of chitosan. Chitosan has free amine group, high reactivity, and can be protonated under acidic conditions. Because of the high crystallinity and the hydrogen bonds between the molecular chains, chitosan exhibits good oxygen-barrier properties. The hydrophilicity of chitosan also provide good grease barrier [63]. In paper coating, chitosan is usually blended with other biopolymers or nanomaterials to improve mechanical and barrier properties of coated paper [64]. Moreover, the positively charged chitosan results in a good affinity and a good retention or adhesion on paper fiber surface [63]. Amino groups of chitosan can be used to form an ionic bond network structure and form complexation with salt ion [65], which further enhances the density of the coating and improves the water barrier property by ten times. The chitosan coating layers have a stable structure and high mechanical strength, but the hydrophilicity of chitosan is detrimental to the water vapor permeability of the coating layer. Therefore, Zhang [66] coated paper with chitosan and beeswax composite coating to study the effect of beeswax and chitosan on coated paper. The experimental results show that composite coating improves the water vapor barrier property of paper by chitosan forming a dense network with paper fiber, while beeswax fills the network gaps. The hydrophobic characteristic of beeswax improves the anti-moisture permeability of the coating layers, and with the increase of drying temperature, beeswax melts well and forms mountain folds on the paper surface, further improving the water vapor barrier property. However, when the content of beeswax is too high, it will penetrate into the paper structure, leading to a poor water vapor barrier performance.

**Figure 8:**
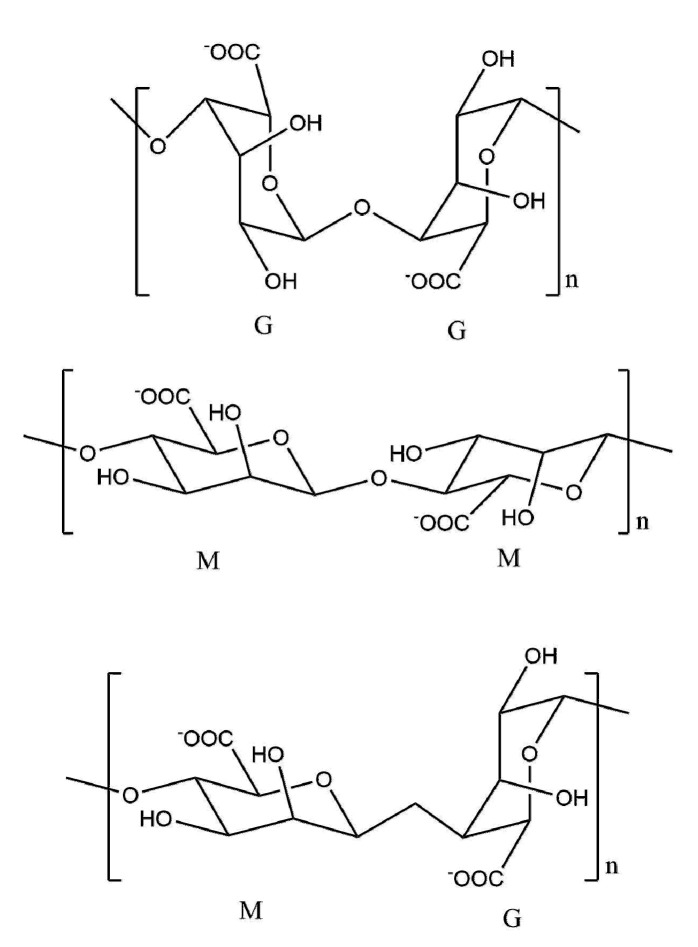
The chemical structures of three types of alginates.

Alginate is a kind of natural polysaccharide that exists in brown algae. It is made up of β-d-mannuronic acid (M) and α-l-guronic acid (G) (Figure 8). In the presence of metal ions, it can form gels [67]. Besides, it has excellent thickening, suspending, and emulsifying properties, and has been widely used in food, medicine [68], pulp and papermaking industry. When sodium alginate is applied as paper coatings, similar to other natural polysaccharides, it can form a dense network structure to improve the barrier property of paper [69]. After being coated by alginate, the paper is densified and the fiber is partially or completely covered by alginate, which results in the decrease of interaction between cellulose fiber and water vapor and the diffusion of water vapor, leading the water vapor transmission rate to reduce by 35–44% [70].

The water vapor transmission rates of different polysaccharide-coated paper are summarized in Table 2. Polysaccharide-based coating can provide paper with good barrier property by densifying fiber network structure, but the hydrophilicity of polysaccharides makes it difficult to decrease water vapor permeability of coatings. As a result, the hydrophobic modification of polysaccharides is needed to improve its performance in barrier coating, and the coating with hydrophobicity can be prepared by modifying polysaccharides with chemicals containing hydrophobic groups.

### 3.2. Water Barrier

Natural polysaccharides have abundant hydrophilic groups (hydroxyl, carboxyl, etc.,) resulting in decreased coating performance under humid environment, so they need to be hydrophobically modified. The presence of hydrophobic functional groups can greatly reduce the wettability of paper surface when hydrophobic polysaccharides form a dense coating layer [71]. Bordenave [72] modified chitosan with dichloromethane and palmitoyl chloride. The results showed that palmitoyl chloride modification made the chitosan coated paper more hydrophobic. The hydrophobic palmitic acid allowed decreasing water vapor transmission rate by 90% and leading to a contact angle above 110°. Hartman [73] found that although GGM with acetyl groups had some hydrophobicity, the introduction of benzyl groups into GGM can significantly improve its hydrophobicity. The water droplet was not adsorbed by the benzylated samples for as long as 10 min even though the samples did not exhibit the highest contact angles.

Different coating methods will change the surface morphology of the coatings, which can also improve the hydrophobic performance of coating layers. Zhang [74] coated the paper with α-cellulose 10-undecylenoyl ester by spray coating method to form irregular nanostructures on the surface of the paper. The hydrophobic properties were maintained under strong acid or alkali condition and it displayed a good durability even after storage for 85 day. The penetration of the hydrophobic cellulose derivative coating solution may swell the paper fiber, reducing the mechanical properties of the paper [71]. However, the reduced mechanical properties are still in the acceptable range, so hydrophobic cellulose derivatives have an application prospect in paper coatings.

Polysaccharides can also act as surfactant and binder to disperse the hydrophobic fillers and fix them onto the paper surface for improved hydrophobicity [75]. Farhat [76] prepared composite paper coating by cross-linking hemicellulose with ammonium zirconium carbonate (AZC) which is a kind of hydrophobic material. With the increase of AZC content in the hemicellulose coating, the water resistance of the coated paper was greatly improved by 44%. However, too many modifications via hydrophobic fillers will reduce the bio-degradability of the coating and may lead to environmental issues.

### 3.3. Oil Barrier

In addition to gas and water barrier, oil barrier is also an important barrier property. Recently, Chi [77] prepared starch-based polyelectrolyte complexes (SPEC) by grafting starch with quaternary ammonium or carboxylate groups. In paper coating, SPEC stabilizes the network structure by ionic complexation, hydrogen bonding, and macromolecular entanglement and improves oil resistance of the coated paper by 93% which was comparable to a typical polyethylene-coated paper board. Besides, the strong ionic crosslinked and entangled starch polymer chains can form a homogenous film structure that resists water penetration. Besides, paper coated with starch matrix with stearic acid showed improved hydrophobicity and grease resistance; both properties are related to the content of fatty acids in the coating [78]. When the content of fatty acid is high, the hydrophobic property is enhanced, but the anti-grease property is reduced.

## 4. Pigment Coating

In pigment coating, inorganic fillers such as calcium carbonate, titanium dioxide, kaolin are filled into the pores between fibers on paper surface to improve its weight, whiteness, glossiness, and flatness for better printing and optical properties [79,80]. The printing performance is often related to the glossiness and porosity of the paper. The size and number of pores can determine the degree of light scattering, thus affecting the glossiness of the paper surface. The coating structure has important impact on the optical properties [81], so the network structure of polysaccharides can improve the optical properties of the paper surface. In paper coating, polysaccharides can change the rheological properties of pigment coating, increase its viscosity, and improve the uniformity and pore structure of the coating layers [23,82]. An appropriate viscosity can make the settling time of inorganic fillers longer, reduce the agglomeration, and increase the dispersion of the fillers [83]. In addition, a charged polysaccharide may be adsorbed on the surface of the filler particles; because of the dual effects of electrostatic repulsion and steric hindrance between filler particles, their flocculation effect is reduced, and their dispersion can be effectively improved [83].

Forming polysaccharide network can help to strengthen the coating layer and the fixing of fillers or pigments onto paper surface. After dissolving, the exposure of the polar groups on oxidized starch increases and the molecules will be entangled with each other. Pigment filler particles are fixed in this tangled network. With the increase of oxidized starch content, the network structure enhanced and the viscosity increased [84]. Some polysaccharides, i.e., CMC and hydroxyethyl cellulose (HEC), can form a network structure and adsorb filler particles, i.e., kaolin, into the structure, which improves the mechanical properties of the coating layers, but it easily causes flocculation when the network is broken, reducing the smoothness and gloss of the coated surface. Kugge [81] studied the effects of CMC and hydroxyethyl cellulose (HEC) on the properties of coatings using calcium carbonate (GCC) as the fillers in pigment coating. CMC does not adsorb the pre-dispersed GCC particles, thus reducing the flocculation of GCC. On the contrary, HEC also adsorbs GCC particles, but its adsorption capacity is very low and would not cause flocculation. When CNF is used as the coating additive instead of CMC, it will not flocculate with the fillers or pigments [82] because of their mutual charged repulsion and the high inter-particle mobility of swollen nanocellulose. Moreover, the performance of CNF coating will not be affected by the type of fillers, because CNF is highly anionic and surrounded by bound water, which is not easy to adsorb the filler particles [23].

## 5. Antibacterial Coating

Different from other natural polysaccharides, chitosan is well-known for its antibacterial activity because of its free amino groups, which can inhibit the propagation of up to 32 types of fungi. The positive charged chitosan can interact with the negative charged cell wall components to change the permeability of cells, or combine with proteins and nucleic acids to interfere with the normal physiological function of bacterial or fungal cells [85]. At present, chitosan has been regarded as a promising coating to produce antibacterial paper [86,87], but the antibacterial effect of chitosan is not stable, which is only active under certain pH conditions. The antibacterial effect of chitosan can be effectively improved by the addition of silver nanoparticles [88], metal oxide, and other antibacterial substances. Li [89] used carboxymethyl chitosan and zinc oxide nanoparticles to make antibacterial coating. The antibacterial effect can be achieved by the interaction between carboxymethyl chitosan and the protein of bacterial cell wall. The cation of carboxymethyl chitosan can flocculate with teichoic-acid and protein in Gram-positive bacterium which destroy the bacterial cell wall and inactivate the bacteria. Besides, the zinc ion released by zinc oxide can combine with the protease of bacteria and kill the bacteria [90]. Zinc oxide has certain antibacterial effects that act synergistically with chitosan, while the antibacterial effect changes with the amount of added zinc oxide.

Except for chitosan, most natural polysaccharides do not have antibacterial properties. But grafting the organic or natural antibacterial agents onto the polysaccharides can also make them obtain antibacterial properties [91,92]. Some organic antibacterial agents (carvacrol, etc.) have strong toxicity and are not suitable for large-scale use. Inorganic antibacterial agents may be a good alternative. The coating can also obtain antibacterial properties by blending inorganic antibacterial agents. For example, Prasad [93] added nano-ZnO into starch coating to prepare coated paper and the releasing of zinc ion can inactivate bacteria. Besides, the nano-ZnO coated paper can absorb ultraviolet light and protect the paper. Antibacterial agent can also be wrapped into hydrophobic polysaccharides to prevent migration and achieve slow release. Furthermore, the antibacterial effect of inclusion is better than surface application. The packing of the antibacterial agents can control their migration and be released by adding active ingredients (soy protein isolates) and maintain their high concentration where and when they needed [94]. In addition, polysaccharide coatings with excellent barrier performance can also control the migration of water, reduce the penetration of oxygen to slow down the growth and development of microorganisms [95].

## 6. Functional Filler-Based Coatings

Based on the chemical properties and structures of polysaccharides and their derivatives, paper coating based on polysaccharides can enhance the mechanical property, barrier property, hydrophobic property, and antibacterial property of coated paper, but it is difficult to give paper conductive, catalytic, fluorescent, and other functional properties. Because of the good affinity between polysaccharides and paper fibers, polysaccharides can be used as adhesives to firmly attach organic or inorganic nano fillers with conductive, catalytic, fluorescent, and other special functions to the paper surface to produce functional paper. Therefore, polysaccharides can be blended with functional fillers to prepare composite functional coatings which could endow paper with special functions.

### 6.1. Conductive Coating

Carbon-based conductive materials such as carbon nanotubes (CNT) and graphene oxide have a large number of polar groups on their surfaces, which are difficult to disperse and easy to flocculate [96], while polysaccharides can effectively disperse these conductive fillers and fix them onto the paper surface [97]. For example, Jabbour [98] dispersed graphite particles and carbon fibers with CMC to prepare conductive paper. CMC could effectively dispersed carbon fiber and avoid agglomeration to improve the homogeneity of coated paper surface. Besides, the polar groups of the conductive fillers are easy to form hydrogen bond with the hydroxyl and carboxyl groups of polysaccharides [99], so the conductive fillers can be anchored tightly on the paper surface through coating process, making the paper a stable conductor. The interaction between polysaccharides and conductive fillers can also be enhanced by the charges distributed on the surface of these polysaccharides. For example, some natural polysaccharides and their derivatives (chitosan [100], CMC [101]) can adsorb conductive fillers by electrostatic interaction, further improving the conductivity of the coating layer. CNT can also intertwine with cellulose fibers to form network structure, and enhance the durability of conductive paper, which have the potential to replace the traditional zinc manganese graphite sheet [102].

Although polysaccharide can well disperse and anchor conductive fillers on paper surface, polysaccharide itself does not conduct electricity, which limits the application of natural polysaccharide in conductive functional coatings. It needs further research to solve this problem, especially by chemical modification.

### 6.2. Catalytic Coating

Natural polysaccharide can have catalytic properties after chemical modification, for example, the hydroxyl groups and halide anions in quaternized chitosan can catalyze the cyclo-addition reaction synergistically [103]. Xylan can also be used as a catalyst to reduce silver ion to silver nanoparticles [104]. However, the catalytic efficiency of polysaccharides is limited. In order to obtain greater catalytic effect for catalytic paper, polysaccharide coatings are used to disperse and anchor catalytic filler such as metal ions (which can be reduced to metal nanoparticles in situ) to paper surface. When the catalytic fillers are embedded in polysaccharide-paper fiber network, coated paper usually has superior catalytic activity and reusability [105].

**Figure 9:**
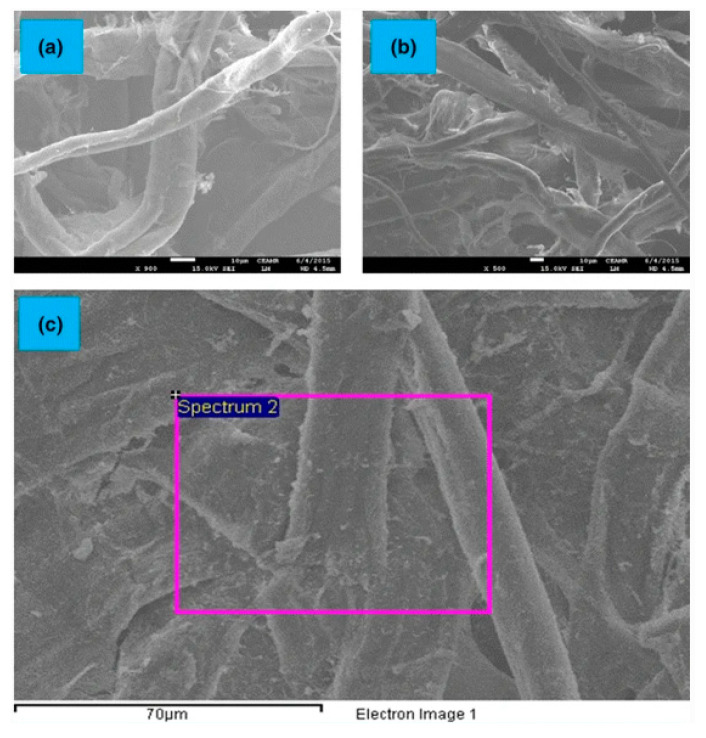
FE-SEM images of (**a**) filter paper (FP), (**b**) filter paper coated with 1% wt chitosan (CH-FP), and (**c**) Cu^0^ loaded CH-FP [109] (Reprinted by permission from: Springer, Cellulose, Synthesis of zero-valent Cu nanoparticles in the chitosan coating layer on cellulose microfibers: Evaluation of azo dyes catalytic reduction, Kamal, T.; Khan, S.B.; Asiri, A.M., 2016).

Since hydroxyl groups and amino groups in chitosan have good coordination and chelation with metal ions, the leach of metal ions could be effectively prevented and the coated paper has a stable catalytic effect [106]. The metal ions can also be reduced by oxidized chitosan with aldehyde groups [107]. It is a promising method to prepare catalytic paper sheets. For example, Ahmad [108] coated chitosan on filter paper, and then loaded it with Ag ions; the catalytic paper for the transformation of nitrophenols to aminophenols was produced after in situ reduction of Ag ions to Ag particles. Because the amine groups of chitosan have the ability to stably form complex with metal ions, the coated paper has good catalytic effect and recyclability, and the transformation rate of the nitrophenols maintained above 90% after its repeated uses. Kamal [109] loaded Cu ion in chitosan coating and coated it on filter paper to catalyze the degradation of methyl orange and Congo red. As can be seen in Figure 9a–b, the surface of cellulose fiber was smooth which indicated the chitosan was coated uniformly. The plenty of bright spots in Figure 9c were due to the Cu nanoparticles adsorbed on the chitosan coating layer. The results showed that the catalytic paper also had good catalytic effect and recyclability. During catalytic reaction, the degradation time of methyl orange and Congo red was shortened by 75%. However, the cost of loading noble metal ions on chitosan is still relatively high, which is not economical to be used in the industry. Another disadvantage is that chitosan is insoluble in water which also limits its large-scale application. It is necessary to reduce the cost of catalyst production and explore a suitable solvent which can dissolve chitosan and load catalyst simultaneously.

### 6.3. Fluorescent Coating

Cellulose can effectively adsorb fluorescent substances because of the strong interaction between cellulose and fluorescein [110], and the CNF with high specific surface area can increase the positive charges on the surface after quaternization modification, which has better adsorption effect on anionic dyes [111]. Moreover, the cellulose network also has a better retention effect on dye molecules, making the fluorescence effect more durable.

Purington [112] first used CNF to adsorb fluorescein isothiocyanate (FITC) and then coated the paper with modified CNF. It is found that fluorescent dyes can be easily adsorbed, and are not easy to dissociate in water, so they exist stably in the CNF network. However, the performance of fluorescent substances is greatly affected by pH, and they cannot exist stably in acidic or base environment. Under acidic condition, CNF may be hydrolyzed, leading to the separation of fluorescein. Furthermore, in the alkaline environment, the deprotonation of FITC may increase its water solubility and cause strong desorption effect. In sum, it is feasible to prepare fluorescent coating by adsorbing fluorescent dyes by cellulose derivative-based coating polymers, but its fluorescence performance is greatly affected by pH, so further improvement is needed.

Patel [113] used 1-pyrenebutyric acid (PyBA) to modify SNPs and prepared pyrene-labeled starch nanoparticles (Py-SNPs). Pyrene was chosen as the fluorescent dye to label SNPs because of its hydrophobicity. Paper coated with Py-SNPs (Py-CFPs) can be used to detect nitroaromatic compounds (NACs) because the NACs are well-known quenchers of fluorescence. When exposed to vapor of NACs, the fluorescence of Py-CFPs quenched to 25% of original value within a short time. Therefore, Py-CFPs is expected to be a candidate material for NACs fluorescence sensor because of its excellent performance and low cost.

## 7. Conclusions and Perspectives

The application of natural polysaccharide in paper coating has many advantages, but there are still some limitations. First of all, the coating properties of polysaccharides are still inferior to that of petroleum-based synthetic polymers, which hinders the industrial application of polysaccharides in paper coating; excessive chemical modification of polysaccharides may cause additional pollution or weaken the bio-degradability of polysaccharides. Second, the polysaccharide-based functional paper coatings rely strongly on the functions of special fillers to endow paper with functions such as conductivity, catalytic, fluorescence, and so on, resulting in less satisfied performance; if polysaccharide-based coating can be endowed with those properties through chemical modification, which may greatly contribute to the production of high performance polysaccharide-based functional coatings. In addition, external environmental factors such as high humidity will greatly affect the performance of polysaccharides coatings. Hydrolysis of polysaccharides will occur under the strong acid and alkali condition, which limits its application in extreme conditions.

Therefore, there are two trains of thought which can solve these problems. First, a mild modification method can be taken to further improve the mechanical properties and reduce the viscosity of polysaccharide-based coatings, making them suitable for modern high-speed paper coating process. Second, it is necessary to explore proper chemical modifications of polysaccharides for special functions. Through the modifications, polysaccharides can be endowed with stable conductive, fluorescent, catalytic, and other properties. More importantly, the polysaccharide coatings should be given the strong resistance to the acid and alkali environment by modification, which will broaden the application range of natural polysaccharides in paper coatings.

## Figures and Tables

**Table 1:** Mechanical properties of polysaccharide-coated paper.

Samples	Coat Weight (g/m^2^)	Tensile Index (Nm/g)	References
Base paper	-	31.9	[28]
CNF	8	40	[46]
CNC	2.56	32.9	[28]
GAX	6	85	[42]
Chitosan—Starch	0.96	79	[47]

**Table 2:** Water vapor transmission rate (WVTR) of polysaccharide-coated paper.

Sample	Coat Weight (g/m^2^)	WVTR (g/m^2^·day)	References
Base paper		490	[49]
CNF	1.6	450	[49]
CNF/CMC	3.3	420	[49]
GGM	1.2	194	[55]
Chitosan	6	276	[69]
Starch	8	347	[59]
Alginate	6.1	386	[69]
